# POSTN-expressing cancer-associated fibroblasts: roles in the tumor microenvironment and clinical implications

**DOI:** 10.3389/fimmu.2026.1952944

**Published:** 2026-10-26

**Authors:** Xiufang Wang, Yongchang Zhao

**Affiliations:** Department of Pharmacy, The Fourth Affiliated Hospital of School of Medicine, and International School of Medicine, International Institutes of Medicine, Zhejiang University, Yiwu, China

**Keywords:** cancer-associated fibroblasts, extracellular matrix, POSTN, tumor immunity, tumor microenvironment

## Abstract

Cancer-associated fibroblasts (CAFs) are among the most abundant stromal components in the tumor microenvironment and play important roles in tumor progression, metastasis, and treatment response. Periostin (POSTN) is an extracellular matrix protein predominantly secreted by CAFs. Accumulating studies have demonstrated that POSTN^+^ CAFs participate in multiple biological processes of tumors, encompassing extracellular matrix remodeling, tumor cell invasion, angiogenesis, and regulation of immune cell function. Through crosstalk with tumor cells and diverse stromal components, POSTN^+^ CAFs mediate the establishment of a pro-tumorigenic microenvironment and can modulate tumor therapeutic responses. Moreover, increased POSTN expression in tumor tissues has been associated with poor prognosis in various malignancies, suggesting the potential utility of POSTN as a biomarker. However, several critical scientific questions remain to be elucidated, including the origin and plasticity of POSTN^+^ CAFs, their relationship with other CAF subsets, and the feasibility of targeting POSTN^+^ CAFs therapeutically. Notably, POSTN^+^ CAFs may represent a reversible fibroblast state rather than an innately determined cell lineage. In this review, we discuss the biological features and functional significance of POSTN^+^ CAFs in cancer, emphasizing their roles in shaping the tumor microenvironment and their potential implications for clinical practice. We further discuss current challenges and future opportunities for targeting this distinct functionally defined fibroblast state, while noting that its identity as a discrete cell population remains to be established.

## 1 Introduction

The tumor microenvironment (TME) is composed of diverse cellular and non-cellular components, including immune cells, endothelial cells, extracellular matrix (ECM), and stromal cells (1, 2). The TME actively regulates tumor initiation, progression, metastasis, and therapeutic responses through complex interactions among its cellular and non-cellular components (3). Among these components, cancer-associated fibroblasts (CAFs) represent one of the most abundant and functionally important stromal populations in solid tumors (4). CAFs are activated fibroblasts that undergo phenotypic and functional changes under tumor-associated signals. Unlike normal fibroblasts, CAFs exhibit enhanced production of extracellular matrix proteins, secretion of cytokines and growth factors, and extensive communication with tumor cells and immune cells. Previous studies have demonstrated that CAFs can promote tumor growth by regulating cancer cell proliferation, invasion, angiogenesis, and immune responses (4, 5). Therefore, CAFs have long been considered important regulators of tumor development.

However, recent advances in single-cell RNA sequencing have challenged the traditional concept of CAFs as a homogeneous population. Increasing evidence indicates that CAFs consist of multiple functionally distinct subsets with different molecular characteristics and biological functions (5). For example, myofibroblastic CAFs (myCAFs) characterized by ACTA2 expression are mainly involved in extracellular matrix deposition and fibrosis, whereas inflammatory CAFs (iCAFs) express inflammatory mediators such as IL-6 and CXCL12 and participate in immune regulation. Antigen-presenting CAFs (apCAFs) have also been identified in several tumor types and exhibit features associated with antigen presentation (5, 6). These findings highlight that understanding specific CAF subsets is essential for deciphering the complex interactions within the TME and developing more precise stromal-targeted therapies.

Periostin (POSTN), also known as osteoblast-specific factor 2, is a secreted matricellular protein belonging to the fasciclin family (7, 8). In normal tissues, POSTN expression is relatively restricted; however, it is frequently upregulated during tissue remodeling, fibrosis, and tumor development. In many solid tumors, POSTN is predominantly expressed by stromal fibroblasts rather than malignant cells, making POSTN a widely used marker for identifying a tumor-promoting CAF population. Accumulating evidence suggests that POSTN^+^ CAFs exert regulatory effects on the extracellular matrix, adjacent tumor cells, endothelial cells, and immune cells primarily via synthesizing and secreting POSTN, which modulates downstream cellular signaling pathways to drive tumor invasion, metastatic dissemination, angiogenesis, and immune modulation (9). Moreover, increasing studies have linked POSTN expression with resistance to chemotherapy and immunotherapy, suggesting that POSTN^+^ CAFs may represent an important stromal component influencing treatment outcomes (9–11). Unlike other well-defined CAF subsets, POSTN^+^ CAFs exhibit substantial plasticity; signals from distinct tumor microenvironments can drive fibroblasts to adopt the POSTN^+^ state or reverse this phenotype (12, 13).

Despite growing interest, several key aspects of POSTN^+^ CAF biology remain unclear. The cellular origin and plasticity of these fibroblasts, their relationship with other CAF subsets, and their potential as therapeutic targets require further investigation. In particular, developing strategies that selectively modulate tumor-promoting POSTN^+^ CAFs without disrupting normal fibroblast functions remains a major challenge. In this review, we summarize current understanding of POSTN^+^ CAFs, focusing on their biological characteristics, roles in tumor microenvironment remodeling, involvement in different cancer types, and potential clinical implications. We also discuss remaining challenges and future directions in POSTN^+^ CAF research. The detailed literature search strategy is provided in **Supplementary Table 1**.

## 2 Biological characteristics of POSTN^+^ CAFs

### 2.1 Biological functions of POSTN in extracellular matrix regulation

POSTN contains an N-terminal EMI domain, four fasciclin-like domains, and a C-terminal region with alternative splicing variants, allowing it to interact with multiple extracellular matrix components and cell surface receptors (12, 14). Once secreted, part of POSTN becomes incorporated into the extracellular matrix, while a proportion enters the circulation and can be measured in peripheral blood. Unlike structural ECM proteins such as collagen, POSTN does not simply provide mechanical support but functions as a regulatory molecule that coordinates interactions between cells and the surrounding matrix.

Under physiological conditions, POSTN is expressed at relatively low levels in most adult tissues but can be induced during tissue remodeling processes, including wound healing, fibrosis, and inflammation. In these contexts, POSTN participates in fibroblast activation, collagen fibrillogenesis, and matrix organization. These biological properties also explain why POSTN expression is frequently increased in tumor tissues, where continuous remodeling of the extracellular environment is required for tumor growth and dissemination. In cancer, POSTN is mainly produced by CAFs, rather than tumor cells themselves. Several studies have demonstrated that POSTN binds to integrin receptors, including integrin αvβ3, αvβ5, and α6β4, thereby activating downstream signaling pathways such as focal adhesion kinase (FAK), Src, and PI3K/AKT signaling. These pathways contribute to enhanced tumor cell motility, survival, and invasive capacity (14, 15).

Importantly, the biological effects of POSTN are highly context-dependent. In normal tissue repair, POSTN contributes to beneficial matrix reconstruction and regeneration. However, in tumors, persistent POSTN production by activated fibroblasts may result in excessive matrix remodeling and create a microenvironment favorable for malignant progression. Therefore, POSTN represents an important molecular link between fibroblast activation and tumor-associated stromal remodeling.

### 2.2 Origin and formation of POSTN^+^ CAFs

The origin of CAFs has been extensively investigated, but the precise developmental trajectory of specific CAF subsets remains incompletely understood. Current evidence suggests that POSTN^+^ CAFs may arise from resident fibroblasts (12, 14). During tumor development, cancer cells and immune cells release various signaling molecules that activate local fibroblasts and induce their transition toward a tumor-associated phenotype. Transforming growth factor-β (TGF-β) is one of the most important factors involved in this process. TGF-β signaling promotes fibroblast activation, enhances ECM production, and induces expression of genes associated with myofibroblast-like and ECM-producing phenotypes, including POSTN (16). In addition to TGF-β, other tumor-associated signals, such as inflammatory cytokines, hypoxia, and mechanical stress, may contribute to POSTN induction. For example, hypoxic conditions within solid tumors can activate hypoxia-inducible factors (HIFs), which regulate fibroblast responses and promote extracellular matrix remodeling (13). Meanwhile, increased matrix stiffness caused by excessive collagen deposition can further reinforce fibroblast activation through mechanotransduction pathways, creating a positive feedback loop that maintains the POSTN^+^ CAF phenotype.

Although fibroblasts represent the predominant source of POSTN in tumors, recent studies suggest that POSTN^+^ CAFs may exhibit considerable plasticity (12). Depending on tumor type, disease stage, and therapeutic pressure, fibroblasts may transition between different functional states. Specifically, nutrient deprivation, inflammatory cytokine-rich microenvironments, and stromal injury induced by chemo- or radiotherapy can reprogram the fibroblast transcriptome and drive fibroblasts toward an ECM-remodeling phenotype (17). Multiple studies have demonstrated that fibroblasts can shift toward a POSTN-high, ECM-remodeling phenotype under sustained hypoxia, TGF-β stimulation, and mechanical stress. Conversely, stress abatement or therapeutic reprogramming can drive POSTN^+^ CAFs back toward a less-activated fibroblast state (13, 16). Therefore, POSTN^+^ CAFs should not be considered a fixed cell population but rather a dynamic functional state shaped by continuous interactions within the tumor microenvironment. POSTN marks an activation state rather than a lineage, and that the term “POSTN^+^ CAFs” is best interpreted from a functional perspective rather than strictly on the basis of ontogenetic origin. The proposed origin and differentiation trajectory of POSTN^+^ CAFs are illustrated in **Figure 1**.

**Figure 1:**
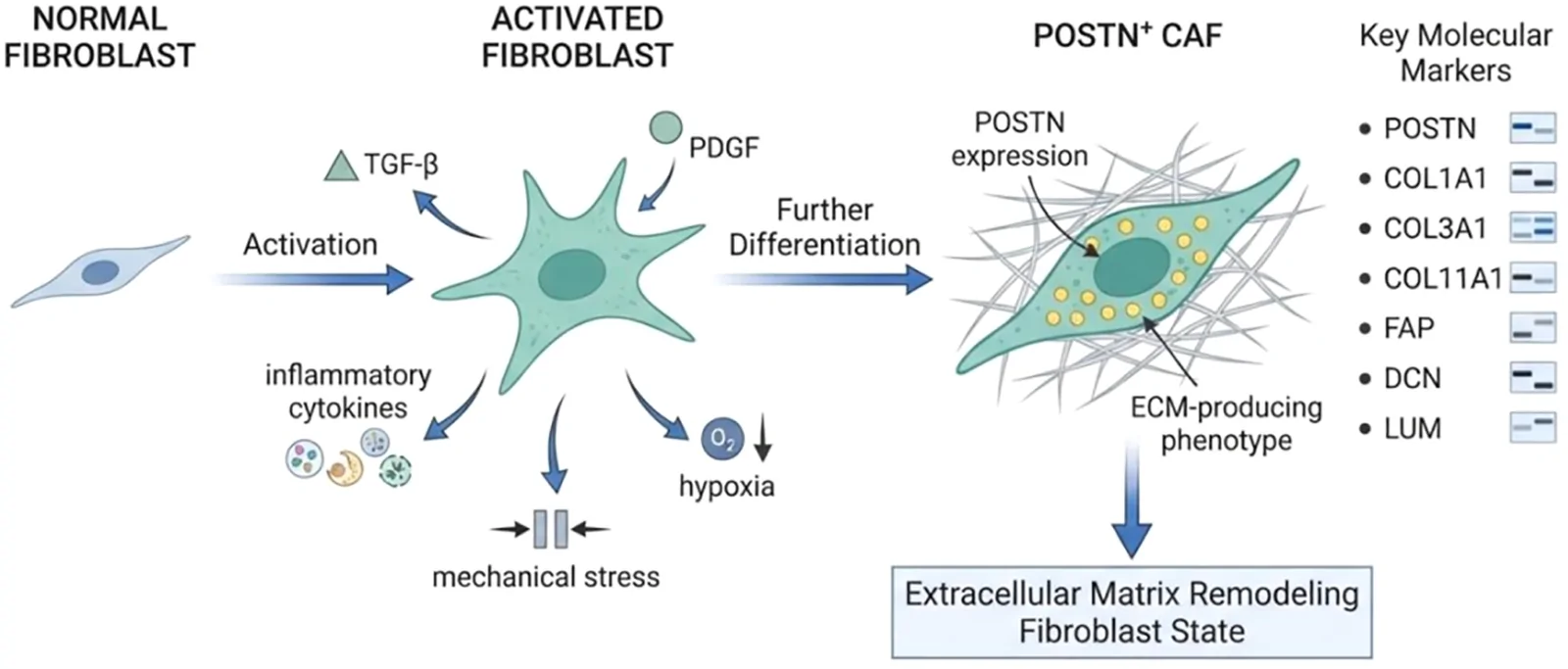
POSTN^+^ CAFs: cellular origin, phenotypic heterogeneity, and spatial distribution. POSTN^+^ CAFs arise from tumor-associated fibroblast activation under microenvironmental cues and represent an ECM-remodeling fibroblast state. This figure summarizes their potential origins, relationship with other CAF subsets, characteristic molecular markers, and spatial distribution within tumors.

### 2.3 Molecular characteristics of POSTN^+^ CAFs and their relationship with other CAF subsets

Single-cell transcriptomic technologies have systematically uncovered the profound heterogeneity of CAFs within the tumor microenvironment and identified functionally distinct CAF states conserved across many solid tumors, mainly including canonical myofibroblastic CAFs (myCAFs), inflammatory CAFs (iCAFs), antigen-presenting CAFs (apCAFs), and COL11A1^+^ CAFs (17–19). Against this backdrop, POSTN^+^ CAFs detectable in multiple human tumors often show POSTN expression overlapping with other CAF markers, especially FAP and COL11A1.

In non-small cell lung cancer, spatial multi-omic profiling revealed that COL11A1^+^ CAFs exhibit a pro-tumor myCAF phenotype with an ECM program encompassing FAP, POSTN and CTHRC1; these cells cooperate with SPP1^+^ macrophages to restrict T-cell infiltration into tumor nests (20). Accordingly, COL11A1^+^ CAFs and POSTN^+^ myCAFs largely describe the same matrix-remodeling functional state, labelled using different gene markers. In contrast, FAP labels a highly heterogeneous CAF pool containing both inflammatory iCAFs and myCAF-like cells. Although a subset of FAP^+^ CAFs co-expresses POSTN, many FAP^+^ iCAFs produce high levels of inflammatory mediators such as IL-6 and CXCL12 with minimal POSTN expression (21), demonstrating only partial overlap rather than complete equivalence between the two populations.

Consistent with this phenotypic overlap, POSTN^+^ CAFs exhibit context-dependent functional heterogeneity across tumor types, spatial locations and disease stages. In pancreatic cancer, POSTN^+^ CAFs drive stromal fibrosis and immune exclusion, forming the characteristic dense, tumor-promoting stroma. In breast cancer, stromal fibroblast POSTN expression facilitates tumor cell invasion and distant metastasis. In colorectal cancer, POSTN^+^ fibroblasts accumulate at invasive tumor margins, where they mediate tumor–stroma crosstalk to support local tumor invasion.

Variations in detection methods, analytical platforms and nomenclature further complicate the definition of POSTN^+^ CAFs. Specifically, single-cell and spatial transcriptomics measure mRNA, whereas immunohistochemistry, multiplex immunofluorescence and imaging mass cytometry detect protein. Coupled with the lack of a unified standard for CAF subtype nomenclature, markers for matrix CAFs differ substantially: the same biological state is termed “mCAF” at the protein level but is commonly referred to as “POSTN^+^ CAF” in transcriptomic studies (22). Therefore, stromal POSTN protein positivity cannot be equated with high abundance of POSTN^+^ CAFs. Multiple confounding factors should be considered when comparing findings across independent studies.

Taken together, current evidence indicates that POSTN^+^ CAFs do not constitute a fixed cellular lineage but are defined by phenotypic and functional characteristics as a dynamically plastic ECM-remodeling fibroblast state governed by the tumor microenvironment.

## 3 Roles of POSTN^+^ CAFs in the tumor microenvironment

The tumor microenvironment is a complex ecosystem in which cancer cells continuously interact with surrounding stromal components. As a specialized fibroblast population, POSTN^+^ CAFs contribute to tumor progression not only through direct regulation of cancer cells but also through remodeling of extracellular matrix, modulation of immune responses, and coordination of interactions among different stromal populations. Increasing evidence indicates that POSTN^+^ CAFs function as important regulators of the tumor niche and influence multiple stages of cancer development (23, 24). The major mechanisms through which POSTN^+^ CAFs regulate tumor progression are summarized in **Figure 2**.

**Figure 2:**
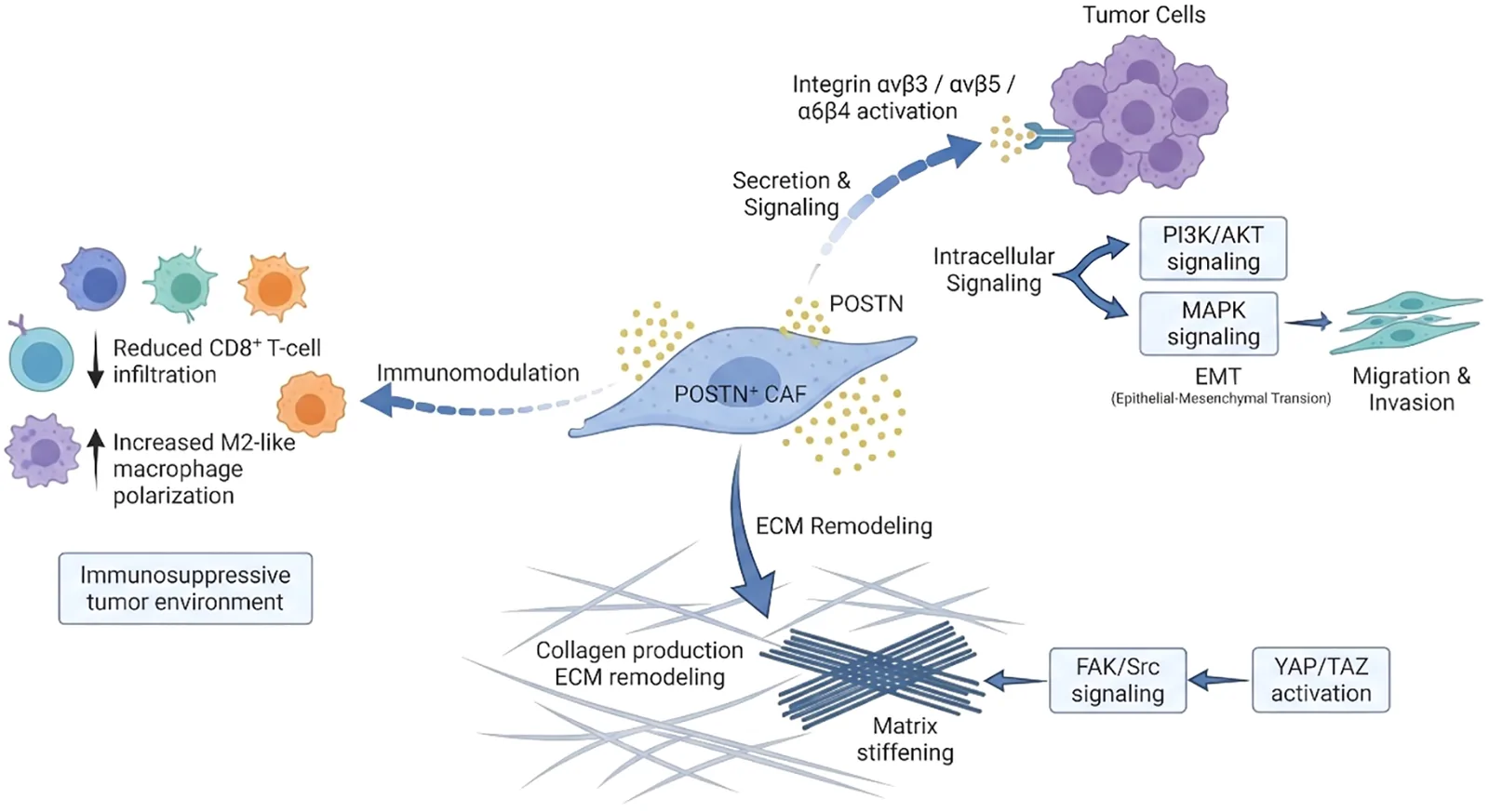
Mechanisms underlying the tumor-promoting functions of POSTN^+^ CAFs. POSTN^+^ CAFs regulate tumor progression through coordinated interactions with tumor cells, extracellular matrix, immune cells, and endothelial cells. This figure illustrates their roles in ECM remodeling, tumor invasion, immune suppression, and angiogenic regulation. In this figure, dashed arrows denote direct POSTN-dependent signaling pathways, while solid arrows represent the general biological functions of CAFs.

### 3.1 POSTN^+^ CAF-mediated extracellular matrix remodeling and tumor invasion

One of the most recognized functions of POSTN^+^ CAFs is their ability to regulate ECM remodeling. POSTN can interact with collagen molecules and facilitate collagen fibril formation, thereby promoting the maturation and stabilization of the tumor-associated matrix (25). Excessive deposition and remodeling of ECM components generate a dense, mechanically rigid stromal microenvironment that further augments the malignant potential of tumors, which is frequently observed in highly fibrotic tumors such as pancreatic cancer (12, 14).

The increased matrix stiffness induced by POSTN^+^ CAFs can further influence tumor cell behavior through mechanotransduction pathways. A stiff ECM promotes activation of integrin-mediated signaling and downstream molecules such as FAK and Src, enhancing cytoskeletal remodeling and cell migration. In addition, mechanical signals from the altered matrix can activate YAP/TAZ signaling in tumor cells, promoting proliferation, epithelial–mesenchymal transition (EMT), and invasive phenotypes. Beyond structural remodeling, POSTN^+^ CAFs also regulate the composition of ECM by affecting matrix degradation and turnover. Through modulation of matrix metalloproteinases (MMPs) and other ECM-associated molecules, POSTN^+^ CAFs facilitate local tissue remodeling, allowing tumor cells to penetrate surrounding tissues and enter vascular or lymphatic systems (15).

What is common across tumor types is that POSTN^+^ CAFs convert the tumor matrix from a structural barrier into a permissive substrate for invasion; the tumor-specific manifestations of this process are discussed in Section 4.

### 3.2 Regulation of tumor cell proliferation, survival, and metastasis by POSTN^+^ CAFs

In addition to remodeling the extracellular environment, POSTN^+^ CAFs directly regulate tumor cell behavior through paracrine signaling. By secreting POSTN and other stromal factors, these fibroblasts establish continuous communication with malignant cells and promote tumor progression. One important mechanism involves POSTN-mediated activation of integrin signaling. Secreted POSTN can bind to integrin receptors expressed on tumor cells, including integrin αvβ3, αvβ5, and α6β4 (26). Activation of these receptors triggers intracellular signaling cascades involving FAK, Src, PI3K/AKT, and MAPK pathways, which enhance tumor cell survival, migration, and resistance to stress (25, 27, 28).

In addition, POSTN^+^ CAFs can promote EMT, a biological process associated with increased tumor cell plasticity and metastatic ability. Periostin secreted by CAFs binds to ITGB1 on the surface of non-small-cell lung cancer (NSCLC) cells, activates the FAK/Src signaling pathway, and triggers EMT-like phenotypic changes, thereby facilitating the invasion and migration of NSCLC cells (29). Such interactions between POSTN^+^ cancer-associated fibroblasts and tumor cells confer survival signals upon tumor cells within hostile microenvironments, enhance tumor-cell adhesion to extracellular matrix constituents, and support local tumor invasion and colonization of distant organs.

Interestingly, POSTN has also been implicated in the formation of pre-metastatic niches. Before tumor cells arrive at distant organs, stromal remodeling and extracellular matrix changes can create a favorable environment for metastatic colonization. POSTN produced by stromal fibroblasts may participate in this process by promoting matrix organization and recruiting additional supportive stromal cells. These findings indicate that POSTN^+^ CAFs actively contribute to tumor progression and metastasis rather than merely reflecting a response to tumor development (23, 30).

### 3.3 Regulation of immune microenvironment by POSTN^+^ CAFs

The dense, collagen-rich matrix assembled by POSTN^+^ CAFs also has immunological implications, given that it physically restricts the access of cytotoxic T cells to tumor nests (31). Spatial co-localization data demonstrate that POSTN^+^/collagen-rich regions are topographically segregated from CD8^+^ T-cell infiltrates, providing correlative evidence for this model. Intervening experiments further reinforce this concept: pharmacological and genetic disruption of the POSTN–matrix axis reduces matrix-mediated immune suppression and restores CD8^+^ T-cell infiltration into tumor cores (32). In addition, the spatial organization of POSTN^+^ CAFs itself determines their immunoregulatory function. Studies have demonstrated that the intermingled distribution of fibroblasts and tumor cells exerts stronger suppression on antitumor immunity than the peripheral barrier formed by fibroblasts. Fibroblast-specific POSTN knockdown reduces TGF-β secretion within this mixed system and restores antitumor immunity in both *in vitro* and *in vivo* models (33).

Beyond structural regulation, POSTN^+^ CAFs actively interact with immune cells to form an immunosuppressive microenvironment. Compared with the physical barrier effect, intercellular signaling crosstalk represents the core mechanism underlying their immunosuppressive function. Ligand–receptor and spatial analyses predict crosstalk in which POSTN^+^ CAFs recruit SPP1^+^ macrophages, and in which macrophage-derived TGF-β1 signals back to POSTN^+^ CAFs through TGFBR1/TGFBR2 and ACVRL1, amplifying stromal programs (34, 35); POSTN secreted by POSTN^+^ CAFs further recruits PD-1^+^ tumor-associated macrophages through integrin–NF-κB signaling, and these macrophages secrete IL-6 that induces tumor-cell PD-L1, thereby suppressing CD8^+^ T-cell activation (36, 37).

POSTN^+^ CAFs also participate in tumor angiogenesis through interactions with endothelial cells. POSTN can directly regulate endothelial cell migration and survival through integrin-mediated signaling, while POSTN^+^ CAFs can indirectly promote vascular remodeling by secreting angiogenic factors such as VEGF and FGFs (37). In addition, their localization around vascular structures suggests that POSTN^+^ CAFs may contribute to the formation of specialized stromal niches that coordinate vascular development and tumor expansion. The interaction between POSTN^+^ CAFs and tumor cells is similarly bidirectional. Tumor-derived factors, including TGF-β, PDGF, and inflammatory cytokines, stimulate fibroblast activation and POSTN expression. In turn, POSTN^+^ CAFs provide ECM components and paracrine signals that enhance tumor cell survival, migration, and invasion. This reciprocal communication is particularly important in tumors with prominent desmoplastic reactions, where stromal remodeling represents a major determinant of disease progression.

Importantly, POSTN^+^ CAFs should not be considered an isolated fibroblast population with fixed characteristics. Increasing evidence indicates substantial overlap between POSTN^+^ CAFs and other ECM-producing CAF subsets, particularly FAP^+^ CAFs and collagen-producing fibroblasts. Depending on tumor type and microenvironmental conditions, different CAF states may undergo phenotypic transitions, highlighting the plasticity of CAF populations. Therefore, a better understanding of POSTN^+^ CAFs requires further investigation of their interactions with other stromal populations and the factors that regulate their functional states. The major molecular features, functional characteristics, and clinical implications of POSTN^+^ CAFs are summarized in **Table 1**. This table only summarizes markers reported in previous studies, which are not specific to POSTN^+^ CAFs and can also be detected in other fibroblast subsets. The characteristics and functional roles of POSTN+ CAFs across different cancer types are summarized in **Table 2**.

**Table 1:** Molecular characteristics, biological functions, and clinical implications of POSTN^+^ CAFs.

Category	Key features	Representative molecules/pathways	Biological or clinical significance
Cellular origin	Mainly derived from activated resident fibroblasts; may also involve other mesenchymal populations (12, 14)	TGF-β signaling, PDGF signaling, inflammatory cytokines, hypoxia-related pathways (13, 16)	Determines CAF activation trajectory and functional plasticity
Molecular phenotype	ECM-producing fibroblast signature	POSTN, COL1A1, COL1A2, COL3A1, COL5A1, COL11A1, DCN, LUM, FAP, THY1 (18, 19)	Defines a matrix-remodeling CAF state across multiple cancers
ECM remodeling	Enhanced extracellular matrix deposition and organization	Collagen assembly, integrin signaling, FAK/Src pathway (25)	Promotes matrix stiffness, tumor invasion, and stromal remodeling
Tumor cell regulation	Paracrine regulation of malignant cell behavior	Integrin αvβ3/αvβ5/α6β4, PI3K/AKT, MAPK, YAP/TAZ signaling (15)	Enhances tumor cell survival, migration, EMT, and metastatic potential
Immune regulation	Modulation of immune cell recruitment and function	CXCL12, immunoregulatory cytokines, macrophage-related signaling (31, 34)	Associated with immune exclusion and immunosuppressive tumor microenvironment
Angiogenesis	Regulation of vascular remodeling	VEGF, FGF, endothelial integrin signaling (37)	Supports tumor vascularization and growth
Biomarker potential	Indicator of tumor-promoting stromal activation	Tissue POSTN expression, spatial POSTN^+^ CAF abundance (18)	Potential prognostic marker and tool for tumor stratification
Treatment resistance	Associated with stromal barriers and altered immune responses	ECM remodeling, immune checkpoint regulation, survival signaling	May predict poor response to chemotherapy or immunotherapy
Therapeutic targeting	Strategies aiming at selective modulation of POSTN^+^ CAFs	POSTN inhibition, integrin/FAK pathway inhibition, CAF reprogramming (7, 38)	Potential approach for precision stromal therapy, but requires specificity

**Table 2:** Characteristics and functional roles of POSTN^+^ CAFs in different cancer types.

Cancer type	Identification method for POSTN^+^ CAFs	Characteristics of POSTN^+^ CAFs	Major biological functions	Clinical cohort size (n)	Clinical relevance	Sample origin/evidence level
Pancreatic ductal adenocarcinoma (PDAC)	scRNA-seq clustering (POSTN^+^, COL1A1^+^, COL3A1^+^, COL11A1^+^, FAP^+^ signature); spatial transcriptomics; bulk RNA-seq signature; IHC/tissue microarray	Enriched ECM-producing CAF population expressing POSTN, COL1A1, COL3A1, COL11A1, FAP; associated with desmoplastic stroma	Promote collagen deposition, matrix stiffening, tumor invasion, immune exclusion, and stromal remodeling	292 PDAC cases (145 upfront surgery, 147 neoadjuvant chemotherapy) (39)	Associated with poor prognosis, fibrosis, limited drug delivery, and potential resistance to immunotherapy	PDA patient samples (39) ^★★★★★^, PDAC tissue microarrays (40) ^★★★^
Breast cancer	Bulk transcriptomic subtyping + WGCNA to identify POSTN as CAF biomarker; scRNA-seq; spatial transcriptomics; IHC on FFPE; serum ELISA	Stromal POSTN expression mainly derived from CAFs; frequently associated with invasive tumor regions	Enhance tumor cell migration, EMT, metastatic niche formation, and tumor–stroma interaction	242 participants in the serum biomarker study (173 breast cancer patients across stages + 69 healthy controls) (41)	High stromal POSTN levels correlate with aggressive disease features and unfavorable survival	Mouse (11) ^★★★★^, serum from patients (41) ^★★★★★^, FFPE tumor specimens (42)
Colorectal cancer	scRNA-seq with differentiation-trajectory inference; integration of 10 spatial transcriptomic datasets; multiplex IHC/mIF	POSTN^+^ CAFs enriched at invasive margins and tumor–stroma interfaces	Regulate ECM remodeling, promote invasion, EMT, and interaction between tumor cells and fibroblasts	34 CRC patients treated with immune checkpoint blockade (scRNA-seq), integrated with 10 spatial transcriptomic datasets (43)	Potential biomarker for aggressive tumor behavior and stromal classification	tumor tissues from patients (43, 44) ^★★★★★^, FFPE tumor specimens (45) ^★★★^
Lung cancer	scRNA-seq on tumor, paired adjacent and normal tissue; Stereo-seq spatial transcriptomics; IHC and multiplexed IHC (mIHC); tissue-derived exosomal RNA sequencing; TCGA-LUAD bulk RNA-seq	ECM-associated CAF subset expressing POSTN and collagen-related genes	Facilitate matrix remodeling, tumor cell migration, and immune suppression	16 NSCLC patients (scRNA-seq); 7 NSCLC patients (Stereo-seq) (18)	May contribute to immune-excluded phenotypes and influence response to immune checkpoint blockade	NSCLC patients’ tissue (18) ^★★★★★^, FFPE LUAD tumor Specimens (46) ^★★★^
Gastric cancer	IHC on an in-house patient cohort; scRNA-seq; public database mining (TCGA); tissue microarray	POSTN-expressing stromal fibroblasts associated with activated CAF phenotype	Promote ECM organization, tumor invasion, and stromal remodeling	278 GC patients on tissue microarrays (47), 40 samples (10 normal stomach, 26 GC, 3 GC peritoneum, 1 normal peritoneum) (48)	Increased POSTN expression associated with tumor progression and poor outcome	Mouse tumor tissue (24, 47) ^★★★^, patients tissue (48) ^★★★★★^, Tissue microarray (47) ^★★★^
Hepatocellular carcinoma	Integration of multi-site scRNA-seq; spatial transcriptomics; snRNA-seq; multiplexed immunofluorescence (mIF); bulk transcriptomics; GSVA scoring	POSTN-producing stromal cells enriched in tumor-associated fibroblast populations	Regulate fibrosis, angiogenesis, and tumor-supportive signaling	91 HCC patients on TMAs (63 treated by resection, paired non-neoplastic liver in 28) (49)	Associated with tumor progression and metastatic potential	FFPE tumor Specimens (49) ^★★★^
Cholangiocarcinoma	scRNA-seq and spatial transcriptomics of tumor core, adjacent non-tumorous tissue and the leading-edge area; IHC on FFPE tissue; ELISA	POSTN^+^ fibroblasts present in highly fibrotic tumor stroma	Contribute to desmoplastic reaction, ECM remodeling, and therapy resistance	116 bile duct carcinoma patients on TMAs (ICC n=19, ECC n=59, GBC n=38) (50)	Potential relevance for stromal-targeted therapies	patients tissue (50) ^★★★★★^, FFPE tumor Specimens (49) ^★★★^
Ovarian cancer	Microarray and bioinformatics analysis of cell lines; TCGA-OV analysis; GEO scRNA-seq; Oncomine and commercial cDNA arrays; IHC	POSTN-high stromal fibroblasts associated with metastatic niches	Facilitate ECM remodeling and peritoneal dissemination	16 paired primary–recurrent high-grade serous OC tissue sets (51)	Associated with advanced disease and poor prognosis	patients tissue (51, 52) ^★★★★★^, Mouse tumor tissue (51, 52) ^★★★★^
Other solid tumors (prostate cancer, glioblastoma, head and neck cancer)	public dataset survival analysis (TCGA, CGGA, Rembrandt); scRNA-seq	Variable enrichment of POSTN-expressing stromal populations	Context-dependent regulation of invasion, matrix remodeling, and immune interaction	Prostate:​ 90 patients who underwent radical prostatectomy (53), Glioblastoma:​ 87 GBM tissue specimens (54), 12 normal brain and 12 glioma tissue samples (8)	Potential prognostic and therapeutic implications require further validation	Human bladder cancer, and prostate cancer cell lines (53) ^★★^, prostate cancer FFPE Specimens (55), glioblastoma FFPE Specimens (8, 54) ^★★★★★^, Mouse tumor tissue (8, 54) ^★★★★^

★ denotes the evidence level; more stars indicate higher evidence confidence. Full definitions are provided in **Supplementary Table 2**.

### 3.4 Pancreatic cancer

Pancreatic ductal adenocarcinoma (PDAC) is one of the tumors most strongly associated with stromal remodeling (56–58). Extensive fibrosis and abundant CAF infiltration are characteristic features of PDAC, making it an important model for studying POSTN^+^ CAF biology. Single-cell transcriptomic studies have identified multiple CAF populations in PDAC, among which ECM-producing fibroblasts expressing POSTN, COL1A1, COL3A1, and COL11A1 represent a major stromal component (9, 39, 59). These fibroblasts are associated with extracellular matrix deposition, tissue stiffness, and tumor progression.

POSTN^+^ CAFs contribute to PDAC progression through coordinated regulation of the tumor stroma. By promoting collagen remodeling and extracellular matrix deposition, these cells contribute to the formation of a dense fibrotic stroma. Meanwhile, their interaction with immune cells helps establish an immunosuppressive microenvironment, while ECM-mediated signaling further enhances tumor cell invasion and metastatic potential (40). Importantly, the dense stroma generated by POSTN^+^ CAFs may also influence therapeutic response. Prior pre-clinical animal studies indicate that the fibrotic environment can impede drug penetration and restrict immune cell infiltration (60), which may partly explain the limited efficacy of conventional chemotherapy and immunotherapy in PDAC. POSTN^+^ CAFs specifically secrete pathological alternatively spliced POSTN isoforms containing exon 21. This isoform binds to HSP70-1a in pancreatic cancer cells, suppresses gemcitabine-induced tumor cell apoptosis, and mediates chemoresistance (40).

### 3.5 Breast cancer

Breast cancer was among the earliest tumor types in which stromal POSTN expression was associated with aggressive disease characteristics (11, 42). In breast cancer, CAF-derived POSTN binds integrin αvβ3, which activates the FAK/PI3K/AKT mechanotransduction pathway to induce EMT and boost local invasion of breast cancer cells; meanwhile, POSTN preserves cancer stem cell properties and promotes tumor colonization in distant organs (61, 62). POSTN^+^ CAFs are also involved in metastatic niche formation, particularly in relation to distant metastasis. By regulating ECM organization and stromal cell recruitment, POSTN may create a favorable environment for tumor cell colonization in metastatic organs. Clinically, increased stromal POSTN expression has been associated with unfavorable prognosis in several breast cancer cohorts, suggesting that POSTN may serve as a potential biomarker for identifying patients with aggressive disease (41).

### 3.6 Colorectal cancer

In colorectal cancer (CRC), POSTN^+^ CAFs are frequently enriched at the invasive margin, where active interactions between tumor cells and stromal components occur (63). On one hand, the POSTN–integrin–NF-κB–PD-1^+^ TAM–IL-6–PD-L1 axis operates in colorectal cancer, where it dampens CD8^+^ T-cell function and establishes an immunosuppressive microenvironment (44, 64). On the other hand, POSTN^+^ CAFs are derived from PI16^+^ and SFRP2^+^ precursor CAFs, whose differentiation is triggered by tumor-secreted GDF15-TGFBR2 signaling and driven by the transcription factors TWIST1 and TCF4. Mature POSTN^+^ CAFs assemble a fortress-like physical stromal barrier that encapsulates tumor cell aggregates, sequestering cytotoxic T cells within the tumor stroma and preventing effector T-cell infiltration into the tumor core. This process further exacerbates resistance to immune-checkpoint inhibitors and collectively drives malignant progression of colorectal cancer (43).

Recent single-cell studies have further demonstrated the presence of distinct fibroblast populations in CRC, with POSTN-expressing fibroblasts representing an ECM-associated CAF subset (43, 45). These cells may contribute to tumor aggressiveness by coordinating matrix remodeling and immune regulation. Given the accessibility of colorectal tumors to tissue sampling, POSTN^+^ CAFs may represent a promising candidate for biomarker development, particularly when combined with spatial and molecular profiling approaches.

### 3.7 Lung cancer

Lung cancer exhibits substantial stromal heterogeneity, and increasing evidence suggests that CAF populations contribute to tumor progression and therapeutic response (18, 46). In NSCLC, POSTN expression is frequently increased in tumor stroma and is associated with aggressive clinicopathological characteristics. POSTN^+^ CAFs promote malignant progression mainly through regulation of the tumor cell–matrix interaction. POSTN^+^ CAFs secrete tenascin-C (TNC) to activate the TNC-integrin signaling axis, which drives EMT in tumor cells (65). Additionally, POSTN^+^ CAFs can release POSTN via exosomes that act on malignant Cancer-AT2 tumor epithelial cells, directly promoting tumor cell proliferation, colony formation, migration and invasion, and facilitating the progression from micro-invasive lesions to invasive lung adenocarcinoma (66).

In addition, POSTN^+^ CAFs may contribute to immune suppression in lung cancer. Several studies using single-cell transcriptomic analysis have identified ECM-producing CAF populations enriched in tumors with reduced immune infiltration (18, 65). These fibroblast populations are associated with increased expression of immunomodulatory molecules and may participate in shaping an immune-excluded microenvironment. The role of POSTN^+^ CAFs is particularly relevant in the context of immunotherapy. Although immune checkpoint inhibitors have improved outcomes for a subset of lung cancer patients, primary and acquired resistance remain major challenges. Stromal remodeling mediated by POSTN^+^ CAFs may represent one factor limiting immune cell access and reducing therapeutic efficacy. Further investigation is needed to determine whether POSTN^+^ CAF abundance can predict immunotherapy response.

### 3.8 Gastric cancer and hepatobiliary cancers

Gastric cancer is characterized by complex interactions between tumor cells and stromal components (48). CAFs are abundant in gastric cancer tissues and have been implicated in tumor invasion, metastasis, and treatment resistance. POSTN expression in gastric cancer stroma has been associated with tumor aggressiveness and unfavorable prognosis. Similar to other malignancies, POSTN^+^ CAFs promote tumor progression through ECM remodeling and regulation of tumor cell behavior. Increased POSTN production enhances matrix organization and provides a supportive environment for tumor invasion (24). Moreover, gastric cancer-associated POSTN^+^ CAFs may participate in immune regulation. By influencing immune cell recruitment and stromal architecture, these fibroblasts contribute to the formation of a tumor microenvironment that favors malignant progression (47).

In hepatobiliary malignancies, including hepatocellular carcinoma and cholangiocarcinoma, stromal remodeling is also an important feature of disease progression (50). In hepatocellular carcinoma, POSTN-producing stromal cells may promote tumor progression by enhancing ECM deposition and activating signaling pathways involved in cancer cell survival. By binding to integrin α5β1 expressed on cholangiocarcinoma cells, POSTN activates the downstream PI3K-AKT-TWIST-2 signaling axis, upregulating effector molecules such as SNAIL-1 and MMP-9, inducing E-cadherin cytoplasmic translocation and cytokeratin-19 down-regulation to drive tumor-cell epithelial-mesenchymal transition, boost migratory-invasive potential, and foster a highly fibrotic microenvironment associated with therapeutic resistance (49, 50, 67). However, compared with pancreatic and breast cancers, the specific contribution of POSTN^+^ CAFs in hepatobiliary tumors remains less well characterized. More studies integrating single-cell sequencing and spatial analysis are required to define their precise functions.

### 3.9 Other solid tumors

POSTN^+^ CAFs have also been reported in several other malignancies, including ovarian cancer, prostate cancer, glioblastoma, and head and neck cancers, although their functions in these tumors remain less well characterized. In ovarian cancer, stromal POSTN expression has been associated with tumor dissemination and peritoneal metastasis, consistent with the important role of ECM remodeling in metastatic spread (51, 52). In prostate cancer, POSTN^+^ stromal cells have been linked to tumor progression and invasive behavior, suggesting their involvement in tumor-stroma interactions during disease evolution (68). Although the cellular composition of glioblastoma differs from that of peripheral solid tumors, POSTN-expressing stromal populations have also been associated with aggressive features and immune regulation (8, 54). Together, these findings indicate that POSTN^+^ CAFs are present across diverse tumor contexts, but their functional significance is largely determined by the specific cellular and stromal environment of each cancer type.

Across multiple solid tumors, POSTN^+^ CAFs exert conserved core functions centered on ECM remodeling, stromal fibrosis, and tumor microenvironment suppression, thereby universally facilitating tumor progression and correlating with poor patient prognosis. Despite these shared properties, POSTN^+^ CAF functions exhibit prominent tumor-specific differences. In desmoplastic pancreatic cancer, POSTN^+^ CAFs predominantly drive dense stroma formation and immune exclusion, establishing a therapy-resistant immunosuppressive microenvironment. Importantly, current evidence is most robust for the pro-fibrotic, pro-invasive, and immune-regulatory roles of POSTN^+^ CAFs in pancreatic and gastrointestinal tumors, supported by abundant single-cell and spatial transcriptomic data. In contrast, the functional characteristics and clinical implications of POSTN^+^ CAFs in breast cancer and other less-studied solid tumors remain less comprehensively defined.

## 4 Clinical implications of POSTN^+^ CAFs

### 4.1 POSTN^+^ CAFs as biomarkers for cancer stratification and outcome prediction

Several techniques are available for the detection and evaluation of POSTN^+^ CAFs in clinical samples (18). Spatial transcriptomics and multiplex immunohistochemistry enable the *in-situ* identification of POSTN^+^ CAFs and the characterization of their distinct spatial distribution patterns. Stromal scoring and composite CAF signatures built from high-throughput transcriptomic data allow bulk quantitative assessment of POSTN^+^ CAF infiltration across large sample cohorts. Circulating POSTN protein in peripheral blood serves as a convenient indirect biomarker. Combined application of these approaches can define stromal phenotypes associated with aggressive tumor behavior, metastatic risk, and therapeutic vulnerability, facilitating precise patient stratification.

Multiple clinical cohort studies have demonstrated that high stromal POSTN expression in tumors correlates with poor clinical prognosis and serves as an independent prognostic factor (69, 70). Single-cell sequencing and clinicopathological analyses further confirm that POSTN^+^ CAFs are enriched in advanced tumors. Elevated POSTN expression is closely associated with tumor aggressiveness, vascular invasion, advanced disease stage, expression of CAF markers, and infiltration of TAMs, and patients with high POSTN expression exhibit worse postoperative outcomes (18, 35, 71). In addition, several clinical studies indicate that increased serum POSTN levels in peripheral blood are significantly correlated with adverse outcomes including distant tumor metastasis and shortened survival, and serum POSTN also acts as an independent prognostic factor (28, 72). However, the clinical interpretation of POSTN remains complex. Conventional measurements based on bulk tumor tissues cannot distinguish whether POSTN originates from fibroblasts, tumor cells, or other stromal populations. Moreover, the abundance and functional state of POSTN^+^ CAFs may vary substantially among different tumor types and anatomical regions.

Despite the availability of these assays, the clinical translation of POSTN^+^ CAF detection faces substantial hurdles. No standardized assay protocols or universally accepted diagnostic cutoffs have been established, and sampling heterogeneity within tumors may introduce measurement bias. It should also be recognized that POSTN detected in tumor tissue or peripheral blood cannot precisely identify its cellular origin and only partially reflects the stromal POSTN pool associated with POSTN^+^ CAFs. Collectively, these methods remain largely confined to research investigations and do not yet meet the requirements for routine clinical implementation.

### 4.2 POSTN^+^ CAFs as potential predictive biomarkers of treatment response

Beyond prognostic significance, preclinical models and correlative analyses of human tissues indicate that POSTN^+^ CAFs can mediate chemoresistance and immune checkpoint inhibitor resistance across multiple solid tumors via stromal remodeling and reprogramming of the tumor immune microenvironment. On the one hand, POSTN^+^ CAFs drive extensive extracellular matrix deposition to form a dense physical stromal barrier that impedes the penetration of chemotherapeutic agents into the tumor parenchyma. They also establish a hypoxic, nutrient deprived microenvironment, which elicits adaptive responses in tumor cells under therapeutic stress and consequently blunts chemotherapeutic responses. On the other hand, POSTN^+^ CAFs contribute to shaping an immune-excluded tumor microenvironment; by reprogramming myeloid immune cells and suppressing the infiltration and effector functions of cytotoxic CD8^+^ T cells, these fibroblasts dampen the anti-tumor efficacy of immune checkpoint inhibitors (40, 64, 65, 67).

Although POSTN^+^ CAF-enriched tumors may represent a stromal phenotype associated with immune exclusion and treatment resistance, direct clinical evidence specifically linking POSTN^+^ CAF abundance with immunotherapy outcomes remains limited (23). Notably, portions of the supporting evidence are derived from *in vitro* experiments, animal models and omics-based correlative analyses. The magnitude of POSTN^+^ CAF-driven resistance exhibits inter-tumor heterogeneity, and large-scale prospective clinical cohorts are still lacking to validate whether POSTN^+^ CAF abundance can serve as an independent predictive biomarker for responses to chemotherapy and immunotherapy (12).

### 4.3 Therapeutic strategies targeting POSTN^+^ CAFs

The pro-tumor properties of POSTN^+^ CAFs have driven the development of CAF targeted therapeutic strategies. Nevertheless, previous attempts to eliminate CAFs as a whole have yielded unexpected outcomes, largely owing to the functional heterogeneity within the CAF pool, in which certain subsets can even restrain tumor progression (38).

Available POSTN-targeted agents have advanced beyond early proof-of-concept studies, with research focusing on neutralizing antibodies that recognize POSTN splice variants. In the syngeneic 4T1 breast cancer model, inhibition of exon-17-containing POSTN in both tumor and stromal compartments suppresses primary tumor growth and lung metastasis (73). The exon-21-specific monoclonal antibody PN21-Ab blocks POSTN-mediated Akt survival signaling and restores cisplatin sensitivity in HSC-3 tongue cancer xenografts (74). In contrast, pan-POSTN neutralization is mainly used for mechanistic validation. In cervical squamous cell carcinoma models, blockade of POSTN or its downstream FAK/Src pathway abrogates POSTN^+^ CAF-induced lymphatic metastasis and reduces the metastatic potential of tumor cells within the lymph-node pre-metastatic niche (75, 76). In ovarian cancer, both POSTN neutralization and gene knockdown inhibit the integrin–NF-κB–TGF-β2 pathway, reducing M2 macrophage recruitment and CAF activation (52). In clear-cell renal cell carcinoma models, pharmacological POSTN blockade reprograms APOE^+^ tumor-associated macrophages, restores CD8^+^ T-cell infiltration into the tumor core, and reduces tumor burden *in vivo* when combined with immune checkpoint blockade (32).In terms of clinical translation, the humanized anti-POSTN antibody PT0101 has entered a phase I/IIa open-label, multicenter clinical trial for advanced solid tumors and HER2-negative recurrent or metastatic breast cancer.

Nevertheless, POSTN^+^ CAF-targeted therapies are not ready for clinical application and have notable limitations. First, available neutralizing antibodies only neutralize POSTN protein irrespective of its cellular source and cannot selectively deplete POSTN^+^ CAFs. Second, POSTN protein neutralization does not necessarily lead to tumor control: in soft-tissue sarcoma models, Postn gene silencing suppresses tumor growth and reprograms myeloid cells, whereas single-agent POSTN neutralization fails to reduce tumor burden (77). Future therapeutic development requires further elucidation of the lineage characteristics, spatial distribution, and functional heterogeneity of POSTN^+^ CAFs to achieve precise stromal modulation. Current therapeutic strategies and future directions targeting POSTN^+^CAFs are summarized in **Figure 3**.

**Figure 3:**
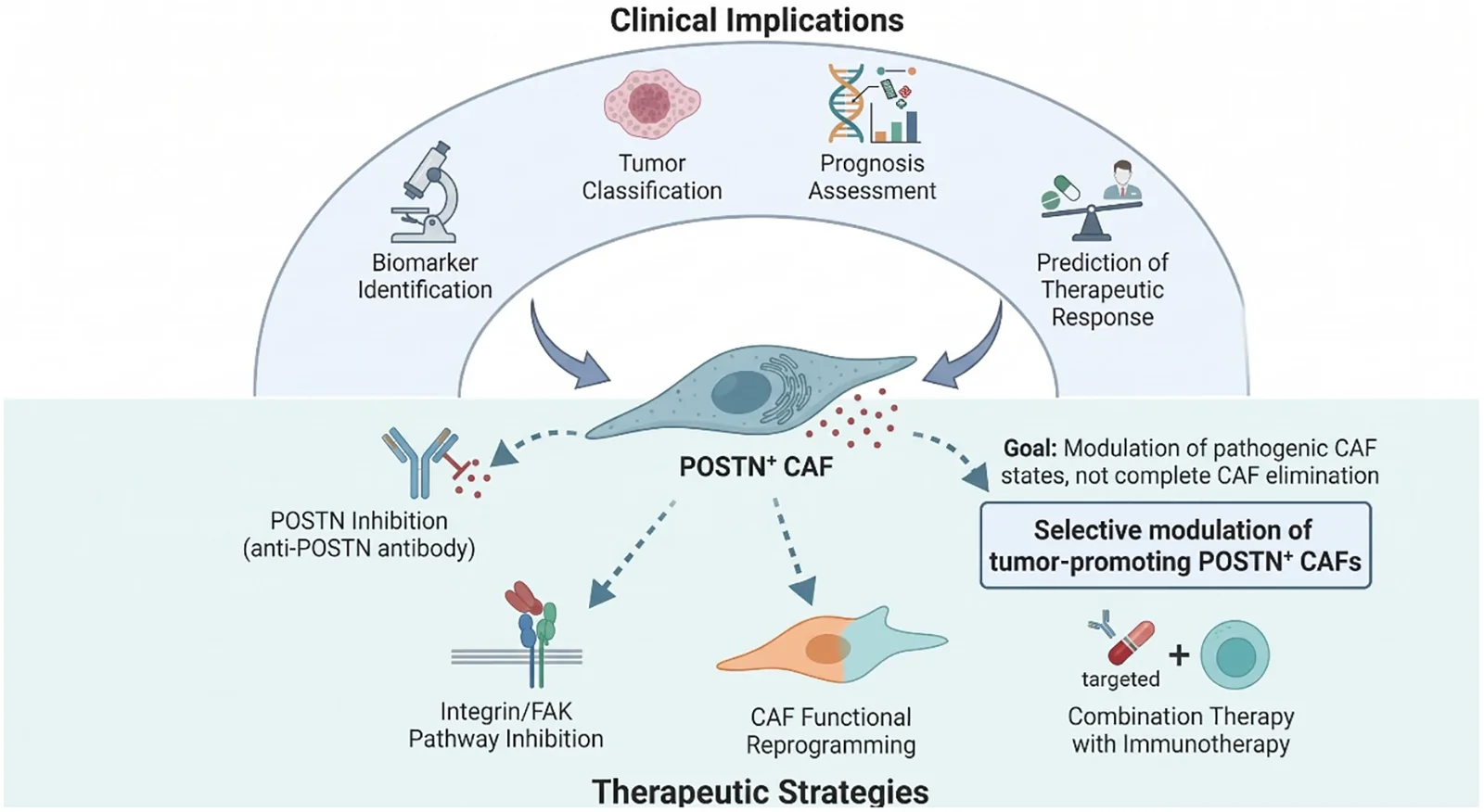
Clinical relevance and therapeutic opportunities targeting POSTN^+^ CAFs. Potential clinical implications of POSTN^+^ CAFs, including their associations with tumor progression, prognosis, and therapeutic resistance, as well as emerging strategies for selective targeting through POSTN inhibition, blockade of related signaling pathways, and CAF functional reprogramming.

## 5 Conclusions and future perspectives

POSTN^+^ CAFs have emerged as an important stromal population in tumor progression and therapeutic response. As discussed above, they are best regarded as a dynamic functional state rather than a fixed lineage, shaped by tumor type, spatial context and microenvironmental cues (18, 38, 78).

Current studies on POSTN^+^ CAFs have several limitations. First, although single-cell and spatial transcriptomics help characterize the biological states and spatial niches of POSTN^+^ CAFs and clarify the relationships among different CAF subsets, transcriptomic data alone cannot define the cellular origin of POSTN^+^ CAFs. The functional heterogeneity of this cell population across different tumor microenvironments still requires further functional validation. Second, interventions targeting the POSTN molecule have confirmed its involvement in multiple biological functions attributed to POSTN^+^ CAFs. However, no study has yet selectively ablated the POSTN-expressing fibroblast population, and the requirement for the population itself therefore remains unproven. From a translational perspective, future studies should focus on defining tumor-promoting POSTN^+^ CAF characteristics and developing strategies for their selective identification and modulation. Integrating single-cell, spatial, and functional approaches will be essential to clarify their clinical relevance and facilitate the development of more precise stromal-targeted therapies.

## References

[1] Li M Yang Y Xiong L Jiang P Wang J Li C . Metabolism, metabolites, and macrophages in cancer. J Hematol Oncol. (2023) 16:80. doi: 10.1186/s13045-023-01478-6 37491279 PMC10367370

[2] Polak R Zhang ET Kuo CJ . Cancer organoids 2.0: modelling the complexity of the tumour immune microenvironment. Nat Rev Cancer. (2024) 24:523–39. doi: 10.1038/s41568-024-00706-6 38977835

[3] Takasugi M Yoshida Y Ohtani N . Cellular senescence and the tumour microenvironment. Mol Oncol. (2022) 16:3333–51. doi: 10.1002/1878-0261.13268 35674109 PMC9490140

[4] Toledo B Zhu Chen L Paniagua-Sancho M Marchal JA Perán M Giovannetti E . Deciphering the performance of macrophages in tumour microenvironment: a call for precision immunotherapy. J Hematol Oncol. (2024) 17:44. doi: 10.1186/s13045-024-01559-0 38863020 PMC11167803

[5] Li P Zhang H Chen T Zhou Y Yang J Zhou J . Cancer-associated fibroblasts promote proliferation, angiogenesis, metastasis and immunosuppression in gastric cancer. Matrix Biol. (2024) 132:59–71. doi: 10.1016/j.matbio.2024.06.004 38936680

[6] Lavie D Ben-Shmuel A Erez N Scherz-Shouval R . Cancer-associated fibroblasts in the single-cell era. Nat Cancer. (2022) 3:793–807. doi: 10.1038/s43018-022-00411-z 35883004 PMC7613625

[7] Park SY Piao Y Jeong KJ Dong J de Groot JF . Periostin (POSTN) regulates tumor resistance to antiangiogenic therapy in glioma models. Mol Cancer Ther. (2016) 15:2187–97. doi: 10.1158/1535-7163.mct-15-0427 27307601 PMC5104278

[8] Wang H Yao L Chen J Li Y Su Z Liu Y . The dual role of POSTN in maintaining glioblastoma stem cells and the immunosuppressive phenotype of microglia in glioblastoma. J Exp Clin Cancer Res. (2024) 43:252. doi: 10.1186/s13046-024-03175-9 39227950 PMC11373117

[9] Wu Y Li S Yu H Zhang S Yan L Guan X . Integrative single-cell and spatial transcriptomics analysis reveals ECM-remodeling cancer-associated fibroblast-derived POSTN as a key mediator in pancreatic ductal adenocarcinoma progression. Int J Biol Sci. (2025) 21:3573–96. doi: 10.7150/ijbs.108618 40520021 PMC12160862

[10] Liu D Tao K Cai C Lin Y Yu R Xiong K . Genomic instability drives POSTN(+) myofibroblasts via STING-WNT axis to promote immunosuppression and PARPi resistance in ovarian cancer. Sci Transl Med. (2026) 18:eady2719. doi: 10.1126/scitranslmed.ady2719 42202048

[11] Ding X Wu Q Du Y Ji MM Yang H Hu Q . CDK16(+) luminal progenitor cell-like tumor cells interacted with POSTN(+) cancer-associated fibroblasts associate with chemo-resistance in breast cancer. Small Methods. (2025) 9:e2401192. doi: 10.1002/smtd.202401192 39930931

[12] Wang H Liang Y Liu Z Zhang R Chao J Wang M . POSTN(+) cancer-associated fibroblasts determine the efficacy of immunotherapy in hepatocellular carcinoma. J Immunother Cancer. (2024) 12:e008721. doi: 10.1136/jitc-2023-008721 39067872 PMC11284881

[13] Qin J Hu S Chen Y Xu M Xiao Q Lou J . Hypoxia promotes malignant progression of colorectal cancer by inducing POSTN(+) cancer-associated fibroblast formation. Mol Carcinog. (2025) 64:716–32. doi: 10.1002/mc.23882 39835715

[14] Yu Y Tan CM Jia YY . Research status and the prospect of POSTN in various tumors. Neoplasma. (2021) 68:673–82. doi: 10.4149/neo_2021_210223n239 34348466

[15] Zhu Z Zhang K Wu W Zhou J Zhang M Chen J . POSTN(+) CAF-derived migrasomes drive hepatocellular carcinoma progression and confer resistance to immunotherapy. Res (Wash D C). (2025) 8:950. doi: 10.34133/research.0950 41132815 PMC12541147

[16] Yue H Li W Chen R Wang J Lu X Li J . Stromal POSTN induced by TGF-beta1 facilitates the migration and invasion of ovarian cancer. Gynecol Oncol. (2021) 160:530–8. doi: 10.1016/j.ygyno.2020.11.026 33317907

[17] Hu Y Ge J Xin M Guo R Wu C Ma W . Stress-adapted cancer-associated fibroblasts as mediators of immunosuppression and therapy resistance. Cancer Lett. (2026) 657:218706. doi: 10.1016/j.canlet.2026.218706 42398852

[18] Chen C Guo Q Liu Y Hou Q Liao M Guo Y . Single-cell and spatial transcriptomics reveal POSTN(+) cancer-associated fibroblasts correlated with immune suppression and tumour progression in non-small cell lung cancer. Clin Transl Med. (2023) 13:e1515. doi: 10.1002/ctm2.1515 PMC10731139 38115703

[19] Kanzaki R Pietras K . Heterogeneity of cancer-associated fibroblasts: opportunities for precision medicine. Cancer Sci. (2020) 111:2708–17. doi: 10.1111/cas.14537 32573845 PMC7419037

[20] Luo H Xia X Huang LB An H Cao M Kim GD . Pan-cancer single-cell analysis reveals the heterogeneity and plasticity of cancer-associated fibroblasts in the tumor microenvironment. Nat Commun. (2022) 13:6619. doi: 10.1038/s41467-022-34395-2 36333338 PMC9636408

[21] Cumming J Maneshi P Dongre M Alsaed T Dehghan-Nayeri MJ Ling A . Dissecting FAP+ cell diversity in pancreatic cancer uncovers an interferon-response subtype of cancer-associated fibroblasts with tumor-restraining properties. Cancer Res. (2025) 85:2388–411. doi: 10.1158/0008-5472.can-23-3252 40215177 PMC12214878

[22] Ohlund D Handly-Santana A Biffi G Elyada E Almeida AS Ponz-Sarvise M . Distinct populations of inflammatory fibroblasts and myofibroblasts in pancreatic cancer. J Exp Med. (2017) 214:579–96. doi: 10.1084/jem.20162024 PMC5339682 28232471

[23] Wang Y Yue B Ni H Chen J Shi R Wang Z . POSTN^+^ cancer-associated fibroblast-CCL3^+^ macrophage crosstalk defines the immune-excluded tumor microenvironment in clear cell renal cell carcinoma. Transl Oncol. (2026) 65:102682. doi: 10.1016/j.tranon.2026.102682 41579565 PMC12860634

[24] Xu X You T Sun Z Bai C Han Q Zhao RC . POSTN+ cancer-associated fibroblasts promote gastric cancer invasion by activating AKT signaling. Stem Cells Dev. (2025) 34:483–91. doi: 10.1177/15473287251393202 41204675

[25] Jin X Deng Q Ye S Liu S Fu Y Liu Y . Cancer-associated fibroblast-derived periostin promotes papillary thyroid tumor growth through integrin-FAK-STAT3 signaling. Theranostics. (2024) 14:3014–28. doi: 10.7150/thno.94207 38773979 PMC11103496

[26] Wasik A Ratajczak-Wielgomas K Badzinski A Dziegiel P Podhorska-Okolow M . The role of periostin in angiogenesis and lymphangiogenesis in tumors. Cancers (Basel). (2022) 14:4225. doi: 10.3390/cancers14174225 36077762 PMC9454705

[27] Ma H Wang J Zhao X Wu T Huang Z Chen D . Periostin promotes colorectal tumorigenesis through integrin-FAK-Src pathway-mediated YAP/TAZ activation. Cell Rep. (2020) 30:793–806:e6. doi: 10.1016/j.celrep.2019.12.075 31968254

[28] Wang S Li Y Li H Yang F Li X Jia D . Periostin promotes papillary thyroid carcinoma progression by modulating mTOR/AKT pathway. Transl Cancer Res. (2026) 15:401. doi: 10.21037/tcr-2025-aw-2452 42305478 PMC13265196

[29] Wu J Wang X Yang Z Zhang Y Lu M Wang L . The CAFs-derived YAP1-periostin axis forms a fibrotic tumor microenvironment to promote metastatic lung tumor growth. J Transl Med. (2026) 24(1):1295. doi: 10.1186/s12967-026-08664-3 42469883 PMC13660253

[30] Chen Y Liu T Zhang S Wu D . A stromal-centered single-cell landscape identifies POSTN(+) fibroblasts as a high-risk stromal subset in hepatocellular carcinoma. Biomarkers. (2026) 22:1–17. doi: 10.1080/1354750x.2026.2678877 42172061

[31] Glabman RA Choyke PL Sato N . Cancer-associated fibroblasts: tumorigenicity and targeting for cancer therapy. Cancers (Basel). (2022) 14:3906. doi: 10.3390/cancers14163906 36010899 PMC9405783

[32] Ge Q Ge S Wang Z Anwaier A Lu J Tian X . APOE(+) macrophages and POSTN(+)CAFs form immune-exclusion niche driving immunotherapy resistance in clear-cell renal carcinoma. Sci Adv. (2026) 12:eaed0733. doi: 10.1126/sciadv.aed0733 42566529 PMC13450211

[33] Naoi Y Inukai Y Izumikawa T Nagasaki J Ishino T Ueda Y . Close spatial interactions between cancer cells and cancer-associated fibroblasts suppress antitumor immunity. Cancer Immunol Res. (2025) 13:1471–84. doi: 10.1158/2326-6066.cir-24-1144 40590673

[34] Ma C Yang C Peng A Sun T Ji X Mi J . Pan-cancer spatially resolved single-cell analysis reveals the crosstalk between cancer-associated fibroblasts and tumor microenvironment. Mol Cancer. (2023) 22:170. doi: 10.1186/s12943-023-01876-x 37833788 PMC10571470

[35] Zhu A Huang J Zhang J Wei W Wang H Lin S . POSTN(+) CAF-SPP1(+) macrophage crosstalk is associated with immune exclusion and poor prognosis in lung adenocarcinoma. J Transl Med. (2026) 24:1074. doi: 10.1186/s12967-026-08746-2 42625168 PMC13495165

[36] Liu X Yao Q Wang Z Wang P Zhang Z Shen Z . POSTN(+) CAFs facilitate gastric cancer peritoneal metastasis by promoting ICAM-1-dependent tumor cell adhesion and CD8(+) T-cell exhaustion. Front Immunol. (2026) 17:1796080. doi: 10.3389/fimmu.2026.1796080 42358949 PMC13291120

[37] De Oliveira Macena Y Cezar MEN Lira CBF De Oliveira L Almeida TN Costa A . The roles of periostin derived from cancer-associated fibroblasts in tumor progression and treatment response. Cancer Metastasis Rev. (2024) 44:11. doi: 10.1007/s10555-024-10233-3 39614015

[38] Chen H Peng Y Ji Z Dong J . SPP1(+) macrophage-POSTN(+) fibroblast-endothelial triad dictates immunotherapy response in bladder cancer. FASEB J. (2026) 40:e71546. doi: 10.1096/fj.202504456RR 41653034

[39] Aref AT Grealey J Pathan M Noor Z Anees A Azad AKM . Mapping the proteomic landscape of pancreatic cancer: prognostic insights and subtype stratification. Cancer Res Commun. (2025) 5:1879–93. doi: 10.1158/2767-9764.crc-25-0229 41003647 PMC12548992

[40] Tsunetoshi Y Sanada F Kanemoto Y Shibata K Masamune A Taniyama Y . A role for periostin pathological variants and their interaction with HSP70-1a in promoting pancreatic cancer progression and chemoresistance. Int J Mol Sci. (2024) 25:13205. doi: 10.3390/ijms252313205 39684914 PMC11641934

[41] Jia L Li G Ma N Zhang A Zhou Y Ren L . Soluble POSTN is a novel biomarker complementing CA153 and CEA for breast cancer diagnosis and metastasis prediction. BMC Cancer. (2022) 22:760. doi: 10.1186/s12885-022-09864-y 35831854 PMC9281047

[42] Lin S Zhou M Cheng L Shuai Z Zhao M Jie R . Exploring the association of POSTN(+) cancer-associated fibroblasts with triple-negative breast cancer. Int J Biol Macromol. (2024) 268:131560. doi: 10.1016/j.ijbiomac.2024.131560 38631570

[43] Zhou Y Yu JH Sui QQ Liao LE Lv RX Liu Y . POSTN(+) cancer-associated fibroblasts forming fortress-like barrier to mediate immunotherapy resistance in colorectal cancer. Cancer Lett. (2026) 657:218683. doi: 10.1016/j.canlet.2026.218683 42309220

[44] Li B Hou S Tian T Yuan S Li A Feng Y . Fibroblast-derived POSTN promotes colorectal cancer progression under high-fat diet by reprogramming fatty acid metabolism in tumor cell. Cell Commun Signal. (2026) 24:91. doi: 10.1186/s12964-025-02618-w 41507973 PMC12870062

[45] Ma Y Wang L Zhang Z Zhao Y Zhao Z Luo R . Comprehensive single-cell transcriptome landscape of metastatic colorectal cancer identifies budding-potential cells and their interactions with cancer-associated fibroblasts. NPJ Precis Oncol. (2025) 9:408. doi: 10.1038/s41698-025-01187-y 41298776 PMC12749751

[46] Sun D Lu J Tian H Li H Chen X Hua F . The impact of POSTN on tumor cell behavior and the tumor microenvironment in lung adenocarcinoma. Int Immunopharmacol. (2025) 145:113713. doi: 10.1016/j.intimp.2024.113713 39672019

[47] To CH Xie F Yu P Wu J Lyu Y Chen B . POSTN is exclusively activated in cancer-associated fibroblasts and leads to unfavorable prognosis of patients with gastric cancer. Mol Clin Oncol. (2025) 23:82. doi: 10.3892/mco.2025.2877 PMC12278709 40692747

[48] Deng C Deng G Chu H Chen S Chen X Li X . Construction of a hypoxia-immune-related prognostic panel based on integrated single-cell and bulk RNA sequencing analyses in gastric cancer. Front Immunol. (2023) 14:1140328. doi: 10.3389/fimmu.2023.1140328 37180146 PMC10169567

[49] Riener MO Fritzsche FR Soll C Pestalozzi BC Probst-Hensch N Clavien PA . Expression of the extracellular matrix protein periostin in liver tumours and bile duct carcinomas. Histopathology. (2010) 56:600–6. doi: 10.1111/j.1365-2559.2010.03527.x 20459570

[50] Zuyin L Zhao L Qian C Changkun Z Delin M Jialing H . Single-cell and spatial transcriptomics delineate the microstructure and immune landscape of intrahepatic cholangiocarcinoma in the leading-edge area. Adv Sci (Weinh). (2025) 12:e2412740. doi: 10.1002/advs.202412740 PMC11831447 39716897

[51] Huang Z Byrd O Tan S Hu K Knight B Lo G . Periostin facilitates ovarian cancer recurrence by enhancing cancer stemness. Sci Rep. (2023) 13:21382. doi: 10.1038/s41598-023-48485-8 38049490 PMC10695946

[52] Lin SC Liao YC Chen PM Yang YY Wang YH Tung SL . Periostin promotes ovarian cancer metastasis by enhancing M2 macrophages and cancer-associated fibroblasts via integrin-mediated NF-κB and TGF-β2 signaling. J BioMed Sci. (2022) 29:109. doi: 10.1186/s12929-022-00888-x 36550569 PMC9784270

[53] Kim CJ Sakamoto K Tambe Y Inoue H . Opposite regulation of epithelial-to-mesenchymal transition and cell invasiveness by periostin between prostate and bladder cancer cells. Int J Oncol. (2011) 38:1759–66. doi: 10.3892/ijo.2011.997 21468544

[54] Li S Chen Y Xie Y Zhan H Zeng Y Zeng K . FBXO7 confers mesenchymal properties and chemoresistance in glioblastoma by controlling Rbfox2-mediated alternative splicing. Adv Sci (Weinh). (2023) 10:e2303561. doi: 10.1002/advs.202303561 37822160 PMC10667838

[55] Nuzzo PV Rubagotti A Zinoli L Ricci F Salvi S Boccardo S . Prognostic value of stromal and epithelial periostin expression in human prostate cancer: correlation with clinical pathological features and the risk of biochemical relapse or death. BMC Cancer. (2012) 12:625. doi: 10.1186/1471-2407-12-625 23273263 PMC3553030

[56] Noguchi S Yamamoto T Nakanishi Y Matsumoto T Shimada Y Shindo K . Prognostic significance of desmoplastic reaction classification and its association with stromal biomarkers in pancreatic ductal adenocarcinoma. Mod Pathol. (2025) 38:100865. doi: 10.1016/j.modpat.2025.100865 40784485

[57] Kamble P Varma T Kumar R Garg P . Computational theranostics strategy for pancreatic ductal adenocarcinoma. Mol Divers. (2026) 30:1837–63. doi: 10.1007/s11030-025-11241-3 40522604

[58] Yu B Shao S Ma W . Frontiers in pancreatic cancer on biomarkers, microenvironment, and immunotherapy. Cancer Lett. (2025) 610:217350. doi: 10.1016/j.canlet.2024.217350 39581219

[59] Yamashita K Kumamoto Y . CAFs-associated genes (CAFGs) in pancreatic ductal adenocarcinoma (PDAC) and novel therapeutic strategy. Int J Mol Sci. (2024) 25:6003. doi: 10.3390/ijms25116003 38892190 PMC11172745

[60] Ho WJ Jaffee EM Zheng L . The tumour microenvironment in pancreatic cancer - clinical challenges and opportunities. Nat Rev Clin Oncol. (2020) 17:527–40. doi: 10.1038/s41571-020-0363-5 32398706 PMC7442729

[61] Naito K Sangai T Yamashita K . CAF-associated genes in breast cancer for novel therapeutic strategies. Biomedicines. (2024) 12:1964. doi: 10.3390/biomedicines12091964 39335478 PMC11428270

[62] Wu T Xiong S Chen M Tam BT Chen W Dong K . Matrix stiffening facilitates the collective invasion of breast cancer through the periostin-integrin mechanotransduction pathway. Matrix Biol. (2023) 121:22–40. doi: 10.1016/j.matbio.2023.05.006 37230256

[63] Kamali Zonouzi S Pezeshki PS Razi S Rezaei N . Cancer-associated fibroblasts in colorectal cancer. Clin Transl Oncol. (2022) 24:757–69. doi: 10.1007/s12094-021-02734-2 34839457

[64] Wei T Wang K Liu S Fang Y Hong Z Liu Y . Periostin deficiency reduces PD-1(+) tumor-associated macrophage infiltration and enhances anti-PD-1 efficacy in colorectal cancer. Cell Rep. (2023) 42:112090. doi: 10.1016/j.celrep.2023.112090 36773295

[65] Wang Z Yan N Sheng H Xiao Y Sun J Cao C . Single-cell transcriptomic analysis reveals an immunosuppressive network between POSTN CAFs and ACKR1 ECs in TKI-resistant lung cancer. Cancer Genomics Proteomics. (2024) 21:65–78. doi: 10.21873/cgp.20430 PMC10756349 38151287

[66] Chen J Zhou C Chen Y Huang K Wang Y Wang J . Exosomal POSTN from cancer-associated fibroblasts drives progression of microinvasive lung adenocarcinoma: insights from single-cell and tissue exosome sequencing analysis. Front Immunol. (2026) 17:1767771. doi: 10.3389/fimmu.2026.1767771 42238572 PMC13226168

[67] Sonongbua J Siritungyong S Thongchot S Kamolhan T Utispan K Thuwajit P . Periostin induces epithelial-to-mesenchymal transition via the integrin alpha5beta1/TWIST-2 axis in cholangiocarcinoma. Oncol Rep. (2020) 43:1147–58. doi: 10.3892/or.2020.7485 PMC7057947 32020235

[68] Halin Bergstrom S Semenas J Nordstrand A Thysell E Wanman J Crnalic S . Morphological heterogeneities in prostate cancer bone metastases are related to molecular subtypes and prognosis. Clin Exp Metastasis. (2025) 42:49. doi: 10.1007/s10585-025-10365-y 40841830 PMC12370827

[69] Xu X Chang W Yuan J Han X Tan X Ding Y . Periostin expression in intra-tumoral stromal cells is prognostic and predictive for colorectal carcinoma via creating a cancer-supportive niche. Oncotarget. (2016) 7:798–813. doi: 10.18632/oncotarget.5985 PMC4808034 26556874

[70] Oh HJ Bae JM Wen XY Cho NY Kim JH Kang GH . Overexpression of POSTN in tumor stroma is a poor prognostic indicator of colorectal cancer. J Pathol Transl Med. (2017) 51:306–13. doi: 10.4132/jptm.2017.01.19 28407462 PMC5445202

[71] Miyako S Koma YI Nakanishi T Tsukamoto S Yamanaka K Ishihara N . Periostin in cancer-associated fibroblasts promotes esophageal squamous cell carcinoma progression by enhancing cancer and stromal cell migration. Am J Pathol. (2024) 194:828–48. doi: 10.1016/j.ajpath.2023.12.010 38320632

[72] Rachner TD Gobel A Hoffmann O Erdmann K Kasimir-Bauer S Breining D . High serum levels of periostin are associated with a poor survival in breast cancer. Breast Cancer Res Treat. (2020) 180:515–24. doi: 10.1007/s10549-020-05570-0 32040688

[73] Shibata K Koibuchi N Sanada F Katsuragi N Kanemoto Y Tsunetoshi Y . The importance of suppressing pathological periostin splicing variants with exon 17 in both stroma and cancer. Cells. (2024) 13:1410. doi: 10.3390/cells13171410 39272982 PMC11394140

[74] Ikebe S Koibuchi N Shibata K Sanada F Shimizu H Takenobu T . Experimental study: The development of a novel treatment for chemotherapy-resistant tongue cancer with the inhibition of the pathological periostin splicing variant 1-2 with exon 21. Cells. (2024) 13:1341. doi: 10.3390/cells13161341 39195230 PMC11353054

[75] Wei WF Chen XJ Liang LJ Yu L Wu XG Zhou CF . Periostin(+) cancer-associated fibroblasts promote lymph node metastasis by impairing the lymphatic endothelial barriers in cervical squamous cell carcinoma. Mol Oncol. (2021) 15:210–27. doi: 10.1002/1878-0261.12837 PMC7782076 33124726

[76] Gillot L Lebeau A Baudin L Pottier C Louis T Durre T . Periostin in lymph node pre-metastatic niches governs lymphatic endothelial cell functions and metastatic colonization. Cell Mol Life Sci. (2022) 79:295. doi: 10.1007/s00018-022-04262-w 35567669 PMC9107454

[77] Xiao JF Ishaya K Ko EY Fowler A Broz MT Guarnerio J . Periostin promotes sarcoma growth by promoting tumor-associated macrophage migration and differentiation. Cancer Res Commun. (2025) 5:2224–35. doi: 10.1158/2767-9764.crc-25-0301 41448855 PMC12740715

[78] Takatsu F Suzawa K Tomida S Thu YM Sakaguchi M Toji T . Periostin secreted by cancer-associated fibroblasts promotes cancer progression and drug resistance in non-small cell lung cancer. J Mol Med (Berl). (2023) 101:1603–14. doi: 10.1007/s00109-023-02384-7 37831111

