# Supplementary material for: POSTN-expressing cancer-associated fibroblasts: roles in the tumor microenvironment and clinical implications
Source: Front Immunol. 2026 Oct 26;17:1952944. doi: 10.3389/fimmu.2026.1952944 (PMC13746210; doi:10.3389/fimmu.2026.1952944)
Supplement: Supplementary file 1 [file Table1.docx]

Supplementary Table 1. Literature‑search strategy

| Database | Retrieval strategy |
| --- | --- |
| Pubmed | ((POSTN) AND (Cancer-associated fibroblast)) AND (2010 [Date - Publication]:2026 [Date - Publication]) OR  (((Periostin) AND (fibroblast)) AND (tumor)) AND (2010 [Date - Publication]:2026 [Date - Publication]) OR  (((Periostin) AND (fibroblast)) AND (tumor microenvironment)) AND (2010 [Date - Publication]:2026 [Date - Publication]) |
| Embase | ('periostin'/exp OR periostin) AND 'cancer associated' AND fibroblast AND [2010-2026]/py OR  ('periostin'/exp OR periostin) AND fibroblast AND tumor AND [2010-2026]/py OR  ('periostin'/exp OR periostin) AND fibroblast AND tumor AND microenvironment AND [2010-2026]/py |
| Web of Science | TS= (Periostin) AND ALL= (Cancer-associated fibroblast) AND PY= (2010-2026) OR  TS= (Periostin) AND ALL= (fibroblast) AND PY= (2010-2026) AND ALL= (tumor) OR  TS= (Periostin) AND ALL= (fibroblast) AND PY= (2010-2026) AND ALL= (tumor microenvironment) |
