# Supplementary material for: POSTN-expressing cancer-associated fibroblasts: roles in the tumor microenvironment and clinical implications
Source: Front Immunol. 2026 Oct 26;17:1952944. doi: 10.3389/fimmu.2026.1952944 (PMC13746210; doi:10.3389/fimmu.2026.1952944)
Supplement: Supplementary file 2 [file Table2.docx]

Supplementary Table 2. Evidence‑weighting consideration

| **Study type** | **Evidence category** | **Priority** | **Interpretation principle** | **Limitations** |
| --- | --- | --- | --- | --- |
| Retrospective patient cohorts (human‑derived clinical evidence) | Clinically‑validated evidence | ★★★★★ (highest) | Serves as primary support for clinical conclusions. Findings from a single cohort are not regarded as definitive; results are synthesized across multiple independent cohorts. | Subject to sample‑size bias, cohort heterogeneity and retrospective confounding; cannot firmly establish causality. |
| In‑vivo animal experiments | Mechanistically‑validated evidence | ★★★★ | Supports potential causal mechanisms; explicitly marked as pre‑clinical observations in the manuscript. | Animal‑model findings cannot be directly extrapolated to human tumor conditions. |
| Single‑cell / spatial transcriptomic datasets | Descriptive / spatially‑associative evidence | ★★★ | Reveals molecular features and cellular spatial crosstalk; used for correlation analysis only, not for standalone causal inference. | Omics data only reflects association without functional perturbation and cannot prove causality. |
| In‑vitro cell‑based experiments | Supplementary descriptive evidence | ★★ (lowest) | Only applied to illustrate basic molecular mechanisms; not used to infer human clinical outcomes. | Lacks intact in‑vivo TME context and cannot recapitulate real tumor biology. |
